# Positive Selection Drives the Evolution of *rhino,* a Member of the Heterochromatin Protein 1 Family in *Drosophila*

**DOI:** 10.1371/journal.pgen.0010009

**Published:** 2005-07-25

**Authors:** Danielle Vermaak, Steven Henikoff, Harmit S Malik

**Affiliations:** 1 Fred Hutchinson Cancer Research Center, Seattle, Washington, United States of America; 2 Howard Hughes Medical Institute, Basic Sciences Division, Fred Hutchinson Cancer Research Center, Seattle, Washington, United States of America; Cornell University, United States of America

## Abstract

Heterochromatin comprises a significant component of many eukaryotic genomes. In comparison to euchromatin, heterochromatin is gene poor, transposon rich, and late replicating. It serves many important biological roles, from gene silencing to accurate chromosome segregation, yet little is known about the evolutionary constraints that shape heterochromatin. A complementary approach to the traditional one of directly studying heterochromatic DNA sequence is to study the evolution of proteins that bind and define heterochromatin. One of the best markers for heterochromatin is the heterochromatin protein 1 (HP1), which is an essential, nonhistone chromosomal protein. Here we investigate the molecular evolution of five HP1 paralogs present in *Drosophila melanogaster*. Three of these paralogs have ubiquitous expression patterns in adult *Drosophila* tissues, whereas *HP1D/rhino* and *HP1E* are expressed predominantly in ovaries and testes respectively. The HP1 paralogs also have distinct localization preferences in *Drosophila* cells. Thus, Rhino localizes to the heterochromatic compartment in *Drosophila* tissue culture cells, but in a pattern distinct from HP1A and lysine-9 dimethylated H3. Using molecular evolution and population genetic analyses, we find that *rhino* has been subject to positive selection in all three domains of the protein: the N-terminal chromo domain, the C-terminal chromo-shadow domain, and the hinge region that connects these two modules. Maximum likelihood analysis of *rhino* sequences from 20 species of *Drosophila* reveals that a small number of residues of the chromo and shadow domains have been subject to repeated positive selection. The rapid and positive selection of *rhino* is highly unusual for a gene encoding a chromosomal protein and suggests that *rhino* is involved in a genetic conflict that affects the germline, belying the notion that heterochromatin is simply a passive recipient of “junk DNA” in eukaryotic genomes.

## Synopsis

Eukaryotic genomes are organized into good and bad neighborhoods. In fruit fly genomes, most genes are found in euchromatin—good neighborhoods that tend to be amenable to gene expression and deficient in selfish mobile elements. Conversely, heterochromatic regions are deficient in genes but chock full of mobile genetic elements, both dead and alive. Cells expend considerable effort to maintain this organization, to prevent bad neighborhoods from exerting their negative influence on the rest of the genome. At the forefront of this organization are the HP1 proteins, which are involved in the compaction and silencing of heterochromatic sequences. First discovered in *Drosophila*, HP1 proteins have been subsequently found in virtually all fungi, plants, and animals.

Most HP1 proteins evolve under stringent evolutionary pressures, suggesting that they lack any discriminatory power in their action. However, a recent paper by Vermaak finds that one of the five HP1 encoding genes in *Drosophila* genomes, *rhino,* bucks the trend and evolves rapidly. *rhino* is predominantly expressed in ovaries, which is where many mobile elements are also active. Their results suggest that *rhino* has been constantly evolving to police a particularly dynamic, novel compartment in heterochromatin with exquisite specificity. Thus, instead of a genomic wasteyard that genes shun and where transposons go to die, heterochromatin now appears to have been shaped by a constant struggle for evolutionary dominance.

## Introduction

Repetitive DNA sequences can constitute large parts of many genomes (approximately 30% in human and fly genomes) and are involved in fundamental cellular processes [1–3]. For example, centromeres in higher eukaryotes consist of large, repetitive regions required for accurate chromosome segregation during each cell division [4]. Heterochromatin flanks the centromere and is also essential for segregation [5–7]. It is composed largely of repetitive DNA and transposable elements and their relics, but can contain genes important for fertility and viability [8,9]. Transcriptionally silent heterochromatin can influence the expression of not only mobile elements embedded in heterochromatin, but also euchromatic genes [6,10–12]. Given the importance of heterochromatin, it is not surprising that perturbation of heterochromatic proteins is associated with cancer and other diseases [13,14].

The study of repetitive heterochromatic DNA lags far behind that of euchromatic regions because heterochromatin is hard to sequence and manipulate experimentally. Even when DNA sequence is available, the underlying evolutionary forces that shape patterns of rapidly changing repetitive sequences and chromosomal architecture are hard to discern. A complementary approach is to study the evolution of protein components that associate with repetitive DNA instead of studying the DNA directly. These protein components have been well studied, especially in *Drosophila* genomes [15–18]. Using a similar strategy, the discovery of positive selection acting on the proteins that bind centromeric DNA has led to the centromere-drive hypothesis that may account for the sequence complexity of centromeres [19–21].

Here, we examine the evolutionary pressures that shape proteins that bind heterochromatic DNA. Heterochromatin protein 1 (HP1) is a ubiquitous component of heterochromatin that is the best available surrogate to study heterochromatin complexity. HP1 was first identified in flies [18,22] and is present in most eukaryotes where it is required for maintenance of most aspects of the heterochromatic state [6,10,11,23]. HP1 consists of a N-terminal chromo domain, a hinge region, and a C-terminal chromo shadow (or simply “shadow”) domain that structurally resembles the chromo domain and mediates homodimerization [16,22,24–26]. The chromo domain binds to histone H3 tails methylated at lysine 9 (H3K9me), a covalent modification associated with heterochromatin maintenance and transcriptional silencing [10,11,27,28] and can directly influence the targeting of HP1 in vivo [29].

Multiple *HP1*-like genes, which may have different functions, can be found in the same genome. In vertebrates, for example, there are at least three *HP1-*like genes (*HP1α, HP1β,* and *HP1γ*) that each encode proteins with distinct localization patterns, despite being about 65% identical [22,30–33]. *Drosophila melanogaster* contains five genes with *HP1*-like domain organization. We undertook a molecular evolutionary study of these *HP1* paralogs in *Drosophila,* aiming to use them as a surrogate for studying heterochromatic DNA evolution. *HP1A* (or *Su[Var]205*) was the first of these to be identified. This *HP1A* gene encodes the prototypic HP1 protein required for heterochromatin maintenance [18,34]. The functions of the other four HP1 proteins are unknown. However, HP1B and HP1C differ from HP1A in their chromatin localization [35], suggesting that their function is not redundant with HP1A. The fourth HP1-like protein, HP1D/Rhino (hereafter referred to as “Rhino”), was discovered in a screen for female sterile mutants [36] whereas we identified the fifth, HP1E, using bioinformatic criteria in this study.

*rhino* mutants display a variety of late-stage eggshell defects, among them the fused dorsal appendages for which the gene was named [36]. Careful characterization of mutant egg chambers revealed several defects [36]. First, nurse cells failed to undergo a higher-order chromatin structure reorganization from a “five-blob” state to a dispersed state at stage 5. Second, although transcript levels of several patterning genes were unaffected, transcripts of key patterning genes such as *gurken* and *oskar* were mislocalized. Furthermore, Gurken protein synthesis was delayed in early egg chambers and germaria, and Gurken protein showed aberrant accumulation in later egg chambers [36]. Unlike other HP1 proteins, Rhino is expressed predominantly during oogenesis [36]. Its unusual expression pattern suggested that the evolutionary constraints on *rhino* might more accurately reflect pressures on heterochromatin in the female germline, relatively free from constraints imposed during somatic expression.

In this report, we show that tagged Rhino protein localizes to distinct foci within the heterochromatic domain of tissue culture cells. Remarkably, we find that all three domains of Rhino show strong evidence of recurrent positive selection. Such positive selection implies that *rhino* is involved in a heritable and recurrent genetic conflict, raising the intriguing possibility that heterochromatin itself might represent a paleontological record of this genetic conflict.

## Results

### HP1 Paralogs in *Drosophila* Genomes

**Figure 1:**
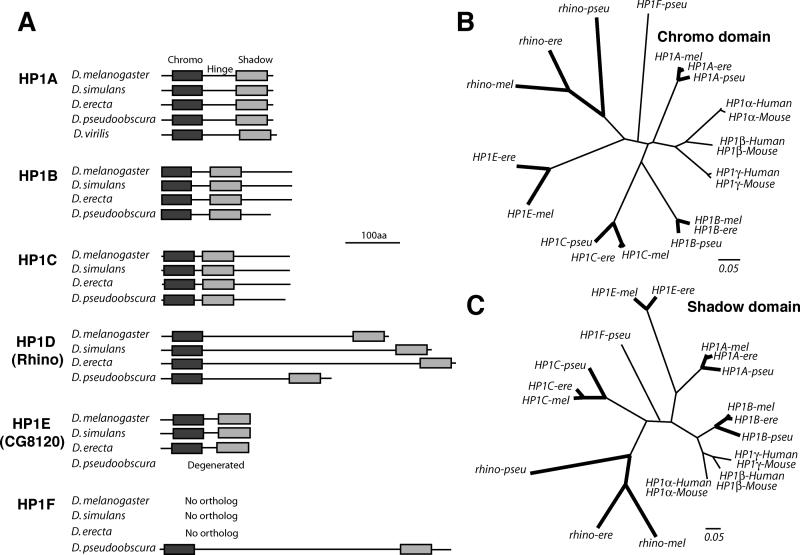
HP1 Paralogs in *Drosophila* (A) Proteins encoded by *D. melanogaster* *HP1*s and selected orthologs (obtained by PCR from syntenic locations) are drawn to scale (indicated at bottom) with a dark rectangle resembling the N-terminal chromo domain and a lighter rectangle the C-terminal chromo shadow domain. The *HP1E* open reading frame is no longer preserved in *D. pseudoobscura,* and *D. melanogaster* does not contain *HP1F*. The hinge regions and N- and C-terminal extensions cannot be aligned between different HP1 types, for example HP1A versus HP1B. HP1D/Rhino contains a very long hinge region that is poorly conserved between species. (B) A neighbor-joining phylogenetic tree based on an alignment of selected HP1 chromo and (C) shadow domains. The monophyletic vertebrate HP1 paralogs are shown for comparison. *rhino* evolution is clearly distinct from vertebrate or other *Drosophila* *HP1*s. *HP1* orthologs between *D. melanogaster, D. erecta,* and *D pseudoobscura* are shown connected by bold branches (*HP1E* is not conserved in *D. pseudoobscura*). The divergence times for *D. melanogaster–D. erecta* and *D. melanogaster–D. pseudoobscura* are approximately 9 and 25 million years respectively, whereas those for mouse–human are approximately 80 million years. Clearly, the *rhino* chromo and shadow domains are far more divergent between these *Drosophila* species than the chromo domains of *HP1A, -B,* and *-C*.

*D. melanogaster* contains five *HP1*-like genes, defined as such because they all encode an N-terminal chromo domain and a C-terminal shadow domain (Figure 1A). Four of these paralogs have been identified in previous analyses [36,37], whereas *HP1E* is newly identified in this report. These paralogs show differences in their conservation across *Drosophila* species. *HP1A, HP1B,* and *HP1C* are highly conserved, even between *D. melanogaster* and the more distantly related *D. pseudoobscura* (Figure 1A). In contrast, *rhino* differs significantly in size and amino acid sequence between *D. melanogaster* and *D. simulans*. In addition, the *HP1E* gene appears to have degenerated in the *D. pseudoobscura* genome, whereas *D. pseudoobscura* possesses *HP1F,* a novel HP1 that the *D. melanogaster* genome lacks altogether.

Among the HP1 paralogs, the *HP1D/rhino* gene appears to be particularly rapidly evolving. In phylogenetic analyses, both the *rhino* chromo and shadow domains appear to have evolved far more rapidly (Figure 1B–C) than their counterparts in other HP1s in *Drosophila* (compare branch lengths between *D. melanogaster, D. erecta,* and *D. pseudoobscura* orthologs, which have bold branches). *HP1E* also appears to evolve rapidly in its chromo domain but is not preserved in *D. pseudoobscura*. Thus, *rhino* appears unique among the *HP1*-like genes in being well conserved yet evolving rapidly. Because *rhino* is evolving so rapidly, orthologs are not likely to be unambiguously identified in other organisms.

### *rhino* Is Expressed Predominantly in Ovaries

**Figure 2:**
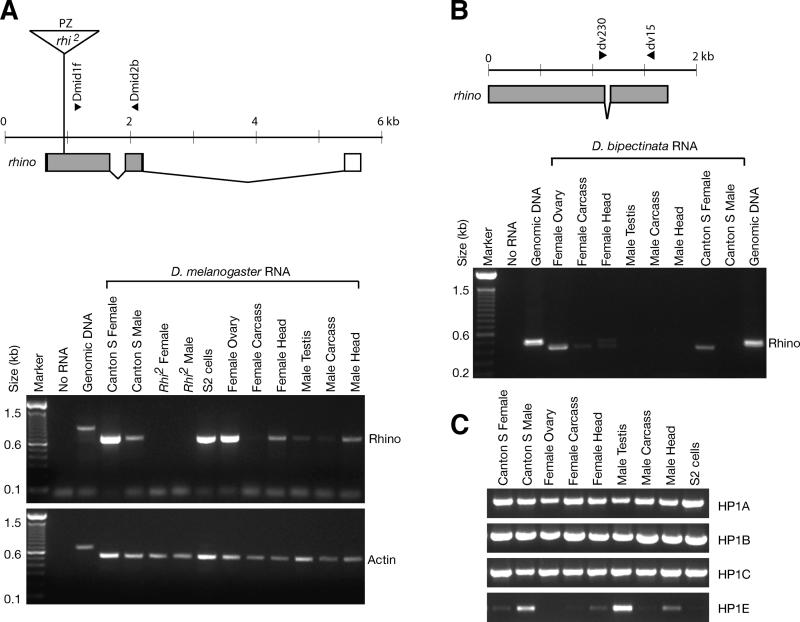
RT-PCR Analyses of the Various HP1 Paralogs (A) The *rhino* gene from *D. melanogaster* is drawn to scale. Exons are boxed (grey fill indicates coding sequence) and lines indicate introns. The position of a *P[lacZ, ry^+^]* (PZ) element in the *rhi^2^* mutant is shown (triangle; not to scale). Dmid1f and Dmid2b RT-PCR primers span the first *rhino* intron. RT-PCR was carried out on roughly equivalent amounts of RNA using a primer set for *rhino* or *actin-42A* (primer sequences in Table S2). Control reactions contained no RNA or *D. melanogaster* genomic DNA. (B) The *rhino* gene from *D. bipectinata* is schematized and primers used for RT-PCR indicated. RT-PCR analysis shows that *rhino* is specifically expressed in ovaries. *D. bipectinata* separated from *D. melanogaster* approximately 13 million years ago. (C) RT-PCR reactions carried out for the other HP1 paralogs in *D. melanogaster*. *HP1A, -B,* and *-C* are ubiquitously expressed in adult tissues whereas *HP1E* expression appears to be predominantly restricted to the male testes.

Previous Northern blot analysis had detected a 1.6 kb *rhino* mRNA in female flies, early embryos, and ovary, but not in male flies and *rhino* mutants [36]. In situ analysis showed that the *rhino* transcript was present both within the germline and somatic cells of the ovary [36]. However, an abundant and much larger band on the Northern blot did not show the same restricted expression pattern. This band was also present in RNA made from *rhi^2^* mutant flies suggesting that it did not contain *rhino* transcript. In order to further delineate the expression pattern of this unusual *HP1* gene, we used RT-PCR to assess the presence of *rhino* mRNA in male or female flies and in different tissues, because it provides a more sensitive assay that complements the previous Northern analysis (Figure 2).

We confirmed the predominant expression of *rhino* in *D. melanogaster* ovaries, although low levels of transcript could also be detected in testis, head, and faintly in carcass, likely below detection limits for Northern analysis (Figure 2A). Endogenous *rhino* transcript was also present in S2 tissue culture cells that were used for our localization studies. Furthermore, the absence of any *rhino* transcript from *rhi^2^* mutant flies by RT-PCR confirms that the large cross-reacting band seen on previous Northern analysis [36] does not contain *rhino* transcript. We have extended this finding to show that the predominant expression of *rhino* is restricted to ovaries in another distantly related species, *D. bipectinata* (Figure 2B). In contrast, we found that *HP1A, -B,* and *-C* genes were abundantly expressed in all gross adult tissues that we examined (Figure 2C). Interestingly, *HP1E* showed an expression pattern restricted predominantly to the male testis, suggesting that two of the five *HP1* paralogs in *D. melanogaster* are each devoted predominantly to testes and ovaries respectively. This may highlight the fact that chromatin structure is likely to be inherently different in somatic versus germline cells, that may have spurred this specialization.

### Rhino Localization in *D. melanogaster* Cells

**Figure 3:**
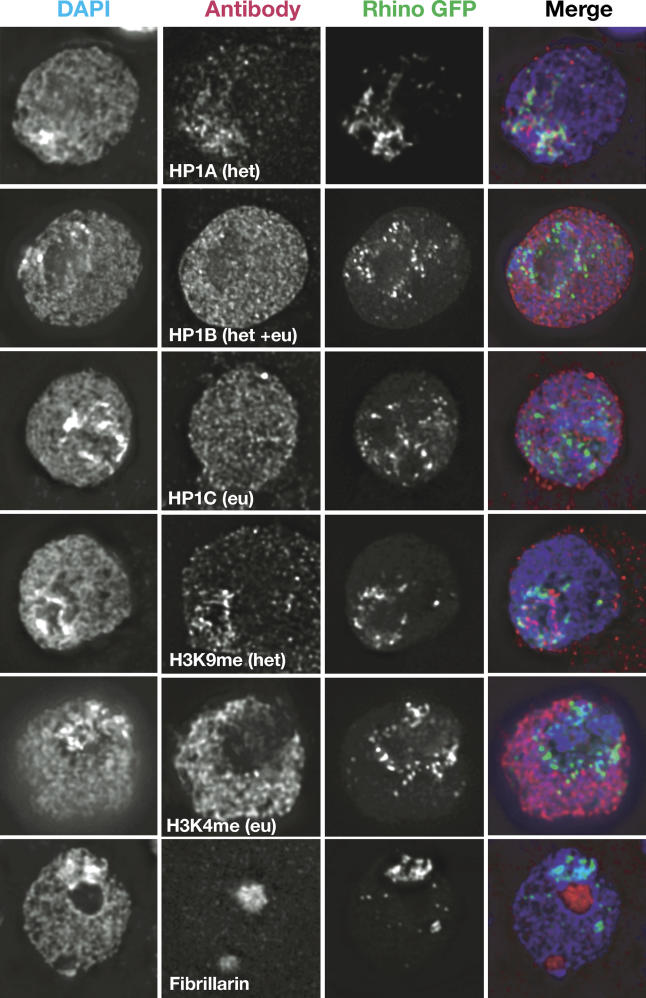
Rhino-GFP Localizes to Distinct Foci in the Heterochromatic Domain A C-terminal GFP fusion protein of *rhino* was transiently expressed in *Drosophila* tissue culture cells (green in merge). Nuclei were stained with DAPI that stains DNA (blue in merge) and antibodies (red in merge) to HP1A, HP1B, HP1C, H3K9me, H3K4me, or fibrillarin (a nucleolar protein). H3K4me stains euchromatin whereas HP1A, H3K9me, Rhino-GFP, and bright DAPI staining all fall within heterochromatin. Rhino-GFP does not overlap with any of the antibody staining patterns, but appears to localize adjacent to HP1A and H3K9me within the heterochromatic domain.

The localization of protein products of three *HP1* genes have been tested so far in *Drosophila* tissue culture cells. Only HP1A was found to localize predominantly to heterochromatin, whereas HP1C localized to euchromatin and HP1B to both euchromatin and heterochromatin [35]. Therefore, we decided to first study the localization pattern of Rhino to determine whether it localized to heterochromatin. *Drosophila* S2 interphase cells have a DAPI-dense staining area that helps demarcate cytological boundaries of heterochromatin, although it is worth noting that DAPI does not stain all heterochromatic DNA, owing to sequence-dependent DNA-binding preference [38]. H3K4me is an excellent cytological marker for euchromatin, whereas H3K9me marks heterochromatin [10,11]. The localization patterns of green fluorescent protein (GFP) fused to HP1A, HP1B, or HP1C and expressed in tissue culture cells were previously shown to be faithful representations of the localization of the endogenous proteins by antibody staining [35]. We therefore expressed *rhino* as a C-terminal GFP fusion protein in *Drosophila* S2 cells, followed by immunostaining with antibodies to HP1A, HP1B, HP1C, or specific modifications of histone H3 (Figure 3) for comparison of localization patterns.

The localization pattern of Rhino-GFP differed from that of HP1A, -B, and -C in interphase tissue culture cells (Figure 3 and Figure S1). Rhino-GFP formed distinct foci that occupied a limited area in the nucleus. These Rhino-GFP foci were located in the heterochromatic compartment as defined by the absence of H3K4me staining. Strikingly, Rhino-GFP also did not directly overlap with common markers of heterochromatin, HP1A or H3K9me, rather appearing interspersed with, or surrounding these signals. Thus, unlike HP1A, we expect that the Rhino chromo domain does not bind H3K9me. The Rhino-GFP localization pattern was not an artifact of GFP-tagging because it was also observed with a Rhino protein that was N-terminally tagged with a biotinylated peptide (Figure S1). We conclude that among HP1 paralogs, Rhino-GFP has a unique localization pattern within the heterochromatic domain in tissue culture cells. Its localization pattern in oocytes is currently unknown.

### Molecular Evolution of *rhino*: Positive Selection of the Hinge and Chromo Shadow Domains

The indication that *rhino* may be a rapidly evolving *HP1* (see Figure 1), its predominant expression in ovaries (see Figure 2), and its interesting cytological localization pattern (Figure 3), led us to investigate its evolutionary history in further detail. Uncovering evolutionary constraints under which different *HP1* genes evolve can provide insight into the evolutionary forces that shape heterochromatin. To study the molecular evolution of HP1 proteins in *Drosophila,* we obtained DNA sequence for HP1 orthologs in the closely related *D. simulans* species (diverged from *D. melanogaster* about 2.5 million years ago) by PCR.

**Figure 4:**
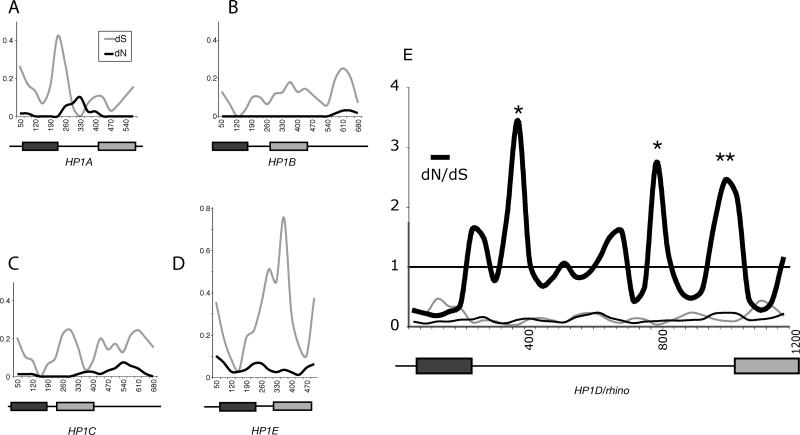
Comparison of *D. melanogaster* and *D. simulans HP1s* (A–E) Different *D. melanogaster* and *D. simulans* HP1 coding DNA sequences were aligned (indels and unalignable sequences were removed) and dN (black line) and dS (grey line) values were calculated using K-estimator [75] with a sliding window of 100 bases and a 35-bp step size. The domain structure of each HP1 is shown schematically and to scale beneath each plot, with the dark rectangle representing the chromo domain and the grey rectangle the chromo shadow domain. For *HP1A,* dN exceeds dS in the hinge region, but dS is very low in these windows. In contrast, for *rhino,* dN is higher throughout and exceeds dS in several windows corresponding to the hinge region (dN/dS values are also plotted for *rhino*). Windows in which statistically significant values for positive selection were obtained (dN/dS > 1, *p* < 0.02), are indicated by asterisks and map to the hinge region.

Rapid evolution of *HP1*s may be attributed to relaxed constraint, allowing sequence changes to accumulate, especially if different gene copies are functionally redundant. Alternatively, amino acid replacement changes may confer a selective advantage, in which case they would be expected to accumulate at a rate higher than expected under neutral evolution (positive selection). To evaluate whether any of the HP1s are undergoing such positive selection between the closely related *D. melanogaster* and *D. simulans* species, we performed a 100-bp sliding window analysis of the number of replacement changes per site (dN) compared to the number of synonymous changes per site (dS) (Figure 4). *HP1B, HP1C* and *HP1E* had dN < dS in all windows, consistent with purifying selection, as expected for structural proteins evolving under strict constraints. To our surprise, we found dN > dS for several windows in the *rhino* gene corresponding to the hinge region of the encoded protein (Figure 4E). We used Monte Carlo simulations in the K-estimator program to show that three of these windows were statistically significant (dN > dS, *p* < 0.02, indicated by asterisks), consistent with positive selection of *rhino* between *D. melanogaster* and *D. simulans*. We also found two windows with dN > dS for the *HP1A-*encoded hinge with borderline significance (*p-*value approximately 0.05), but further detailed analysis including a population study of *D. melanogaster* and *D. simulans,* and dN/dS comparisons among several other pairs of closely related *Drosophila* species (D. Vermaak, H. S. Malik, unpublished data) led to the conclusion that there was no positive selection of *HP1A*. Thus, no other *HP1* homolog other than *rhino* showed any evidence of positive selection, suggesting that *HP1D/rhino* is again unique in this respect, not just among *Drosophila* *HP1* paralogs, but also among all *HP1* genes identified so far.

**Figure 5:**
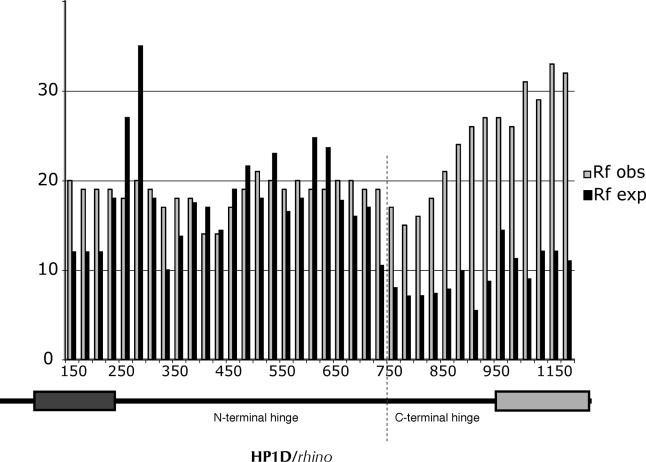
Population Genetics of HP1D/*rhino* between *D. melanogaster* and *D. simulans* Replacement changes that have been fixed between *D. melanogaster* (17 strains) and *D. simulans* (11 strains) (Rf obs [observed], open bars) were calculated with a 300-nucleotide sliding window, 25-nucleotide step size. The number of expected replacement changes for each window (Rf exp; solid bars) were calculated from the neutral expectation of the McDonald-Kreitman test (Rf:Sf ≈ Rp:Sp). Rf obs exceeds Rf exp in the C-terminal part of *rhino* (the C-terminal part of the hinge and the shadow domain as shown beneath), consistent with positive selection (also see Table 1). The chromo and chromo shadow domains are represented by dark and light rectangles, respectively.

Sliding window dN/dS analyses suggest that *rhino* is subject to positive selection. To follow up on this initial observation, we undertook a more detailed study in *D. melanogaster* and *D. simulans*. We used PCR to obtain *rhino* sequence from 17 strains of *D. melanogaster* and 11 strains of *D. simulans*. DNA sequence changes were categorized as replacement (R) or synonymous (S) (Table S1). Changes were further classified as either fixed between species (f) or polymorphic within species (p) (Table S1). Under a neutral evolutionary model, the ratio of replacement to synonymous changes that have been fixed between species (Rf:Sf) is expected to be roughly the same as the ratio for polymorphic changes (Rp:Sp) (McDonald-Kreitman test) [39]. We did not find a significant deviation from neutrality when the entire *rhino* sequence was considered (Table 1, entire coding region, *p* = 0.13). However, a sliding window analysis clearly showed that the observed fixed replacement changes far exceeded those expected under neutral evolution in the C terminal part of the protein (Figure 5). Indeed, the shadow domain had a highly significant deviation from neutrality (*p* < 0.01), suggesting that this domain has been subject to strong positive selection (Table 1, shadow). We used parsimony to assign each DNA sequence change within the shadow domain to either the *melanogaster* or *simulans* lineage by polarizing the changes to outgroup species *D. teissieri* and *D. yakuba* (Table S1, changes in the *melanogaster* lineage [m] or *simulans* lineage [s]). We concluded that the shadow domain has been subject to positive selection in the *D. simulans* lineage (Table 1, shadow *D. simulans* only, *p* < 0.05), but there were not enough polymorphisms to reach a similar conclusion for the *D. melanogaster* lineage despite a strong Rf:Sf ratio. Although the complete hinge alone does not reject neutrality, separating the long hinge domain into N- and C-terminal segments suggests that the C-terminal region of the hinge, abutting the shadow domain, has been subject to positive selection (*p* < 0.01). We could not determine whether the positive selection in the hinge was lineage specific because of ambiguity in the alignment with outgroup species. Despite this strong signal for positive selection, we were unable to detect evidence of recent adaptive “sweeps” using Fu and Li [40] or Tajima [41] tests, suggesting that any such sweeps were not recent enough to result in standing single polymorphisms. Thus, both the hinge and shadow domains of the protein encoded by *rhino* show strong evidence for relatively old episodes of positive selection between the *D. melanogaster* and *D. simulans* lineages.

**Table 1:**
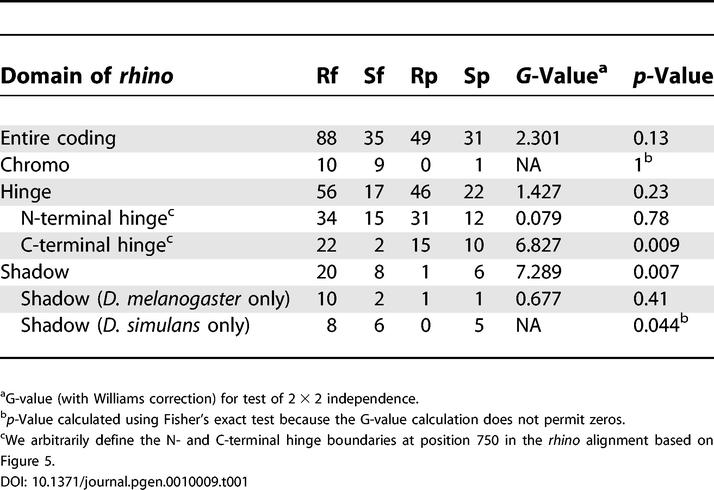
McDonald-Kreitman Test of *rhino* in *D. melanogaster* and *D. simulans*

^a^G-value (with Williams correction) for test of 2 × 2 independence.

^b^ *p-*Value calculated using Fisher's exact test because the G-value calculation does not permit zeros.

^c^We arbitrarily define the N- and C-terminal hinge boundaries at position 750 in the *rhino* alignment based on Figure 5.

### *rhino* Evolution in Other *Drosophila* Species

**Figure 6:**
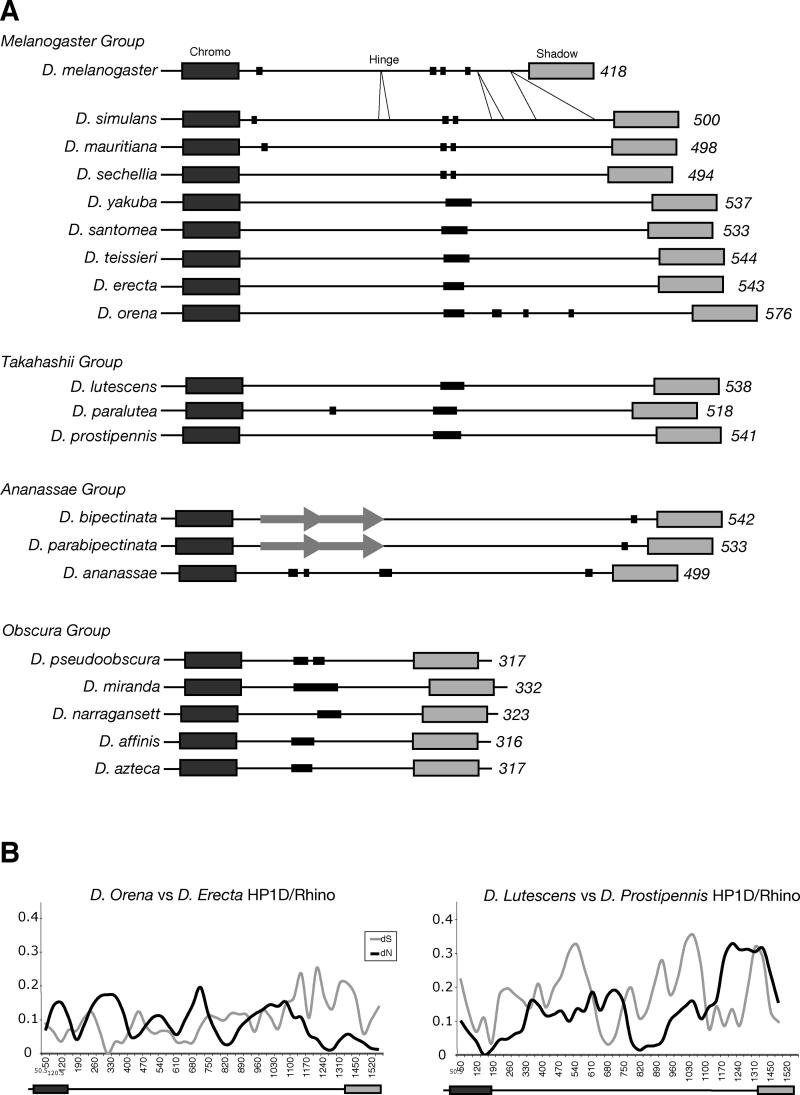
Positive Selection of *rhino* in 25 Million Years of *Drosophila* Evolution (A) *rhino* was PCR amplified and sequenced from the indicated *Drosophila* species. Predicted protein sequences are drawn to scale with amino acid length shown on the right. The chromo and chromo shadow domains are relatively conserved and are indicated by the large dark and light rectangles, respectively. The hinge regions are rapidly evolving. They differ dramatically in size and sequence and cannot be aligned between different species groups (indicated on the left) and sometimes not even within the same species group, for example the *D. bipectinata* versus *D. ananassae* hinge. Within the *melanogaster* species group, *D. melanogaster rhino* appears to have undergone large deletions up to 50 codons in the hinge region compared with its closest relative *D. simulans* (indicated by slanted lines). These deletions are adjacent to the adaptively evolving hinge region identified between *D. melanogaster* and *D. simulans*. A 58 amino acid duplication present in the *ananassae* species group is indicated by grey arrows. Thin black rectangles indicate runs of serine ranging between 70% and 100% serine. (B) dN and dS calculations for *rhino* from alignments of two pairs of closely related species from the *melanogaster* and *takahashii* species groups show multiple windows in which dN exceeds dS, indicative of positive selection.

Is the positive selection of *rhino* limited to the *melanogaster* species group? To address this question, we identified *D. pseudoobscura* *rhino* by synteny with *D. melanogaster;* *rhino* is contained within an intron of another gene in both species. We used RT-PCR to confirm the predicted splice sites for *rhino* from the *obscura* species group. *D. pseudoobscura* *rhino* is very different in length (317 vs. 418 encoded amino acids) and sequence from *D. melanogaster* *rhino* (see Figure 1). In fact, the hinge region of the Rhino protein is changing so rapidly that it is unrecognizable in a BLAST comparison between *D. melanogaster* and *D. pseudoobscura* (*e-*value > 1,000). To trace the evolution of *rhino* beyond the *melanogaster* species group, we obtained *rhino* sequence from intervening species between *D. melanogaster* and *D. pseudoobscura*. Despite the evolutionary distance of *D. melanogaster* from *D. pseudoobscura*, we could identify noncoding conserved sequences both upstream and downstream of the *rhino* gene, allowing us to design primers to amplify *rhino* from 12 additional *Drosophila* species, shown schematically in Figure 6A.

We find that *rhino* is evolving at an unprecedented rate for an *HP1.* The hinge regions cannot be unambiguously aligned between different species groups or in some cases not even within the same species group. We did not detect any significant similarity of the Rhino hinge regions to other proteins or motifs, yet all the hinge regions share certain sequence features, most noticeably long runs of serines as well as proline- and glutamine-rich sequences (Figure 6A). In some instances, we found clear evidence of positive selection (dN/dS > 1) for alignable segments of hinge regions of closely related pairs, *D. yakuba* versus *D. teissieri*, *D. erecta* versus *D. orena*, *D. lutescens* versus *D prostipennis*, *D. bipectinata* versus *D. parabipectinata,* and *D. pseudoobscura* versus *D. miranda* (representative examples shown in Figure 6B). Our ability to detect instances of dN/dS > 1 within the hinge region for multiple species pairs within a small sampling of *Drosophila* species suggests that positive selection of the hinge is a common feature in *rhino* evolution.

### Positive Selection of *rhino* Chromo Domain

Phylogenetic analyses (see Figure 1B and 1C) suggested that not just the hinge region, but also the chromo and shadow domains of Rhino are diverging more rapidly than similar domains of other HP1s. For the hinge and shadow domains, we have already presented evidence that this rapid evolution is not due to lack of selective constraint, but rather due to positive selection. However, we were unable to detect positive selection within the chromo domain using dN/dS or McDonald-Kreitman tests, nor were we able to detect significant evidence of an adaptive sweep using standard tests (Fu and Li [40], Hudson-Kreitman-Aguade [42], Tajima's D [41]). We reasoned that it may be hard to detect positive selection of the chromo domain because the majority of its codons are likely to be functionally constrained and therefore under purifying selection. However, such purifying selection may be masking positive selection of a small number of codons within the chromo domain. We therefore used a codon by codon maximum likelihood test, PAML [43], to ask if we could detect any codons that have been under repeated, strong positive selection.

**Figure 7:**
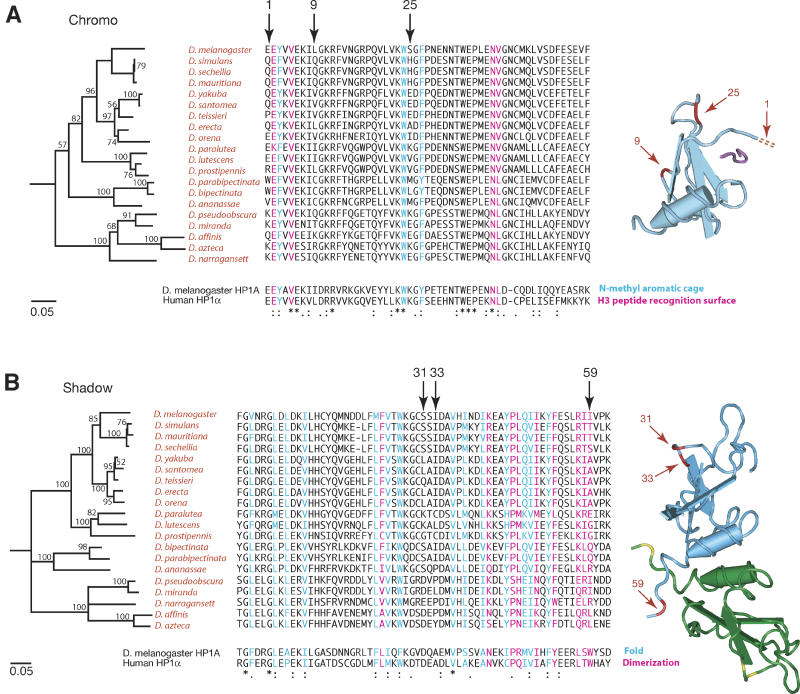
Positive Selection of the Chromo and Chromo Shadow Domains of *rhino* (A) An amino acid alignment of the chromo domain of different *Drosophila* species is shown with the distantly related *HP1A* and human *HP1α* chromo domains for comparison. The neighbor-joining tree based on this alignment (shown on the left) recapitulates known *Drosophila* phylogeny. Amino acids of the *HP1A* chromo domain that are involved in binding to H3K9me are color coded: Blue amino acids form an aromatic cage that recognize K9me, and pink amino acids form a complementary surface for recognition of the H3 peptide [45]. The corresponding DNA sequence alignment was used in a PAML analysis. Three codons that have been under repeated and strong positive selection are indicated by arrows. The corresponding positions (red) are indicated on the known structure of the *Drosophila* *HP1A* chromo domain (light blue) bound to H3K9me (purple) [45]. (B) Amino acid alignment of representative chromo shadow domains of *rhino* orthologs from *Drosophila*. The neighbor-joining tree based on this alignment also recapitulates *Drosophila* phylogeny. Amino acids of mouse *HP1β* known to be involved in dimerization are shown in pink and those required for the shadow fold in blue [46]. We use arrows to indicate codons identified as being under positive selection by our PAML analysis. Corresponding positions of the mouse *HP1β* chromo shadow domain are indicated (red) on one of the shadow domains (light blue) of the dimer [46]. These positions are shown in yellow on the other shadow domain (green).

We used a DNA sequence alignment of the *rhino* gene corresponding to the encoded chromo domain from different *Drosophila* species. The corresponding amino acid sequence alignment is shown in Figure 7A. We note that a tree based upon this amino acid alignment is in agreement with the accepted *Drosophila* phylogeny [44], suggesting that we are considering strict orthologs. Remarkably, models that allow codons to evolve under positive selection (M8 and M2) fit the data significantly better than associated models that do not permit positive selection (M7 and M1) (*p* < 0.001 in all cases, Table 2). Just a few codons account for this positive selection. In particular, three codons repeatedly show highly significant posterior probabilities (1E, 9L, and 25S in Table 2; arrows Figure 7A). The Rhino chromo domain structure is likely to be similar to that of known HP1 chromo domains, so we show the likely positions of the three adaptively evolving amino acids of the Rhino chromo domain on the known structure of *Drosophila* HP1A chromo domain bound to H3K9me peptide (Figure 7A; [45]). Position 1 is in close proximity to the groove that binds the methylated peptide, suggesting that this amino acid may be driven to adapt to a constantly changing substrate of Rhino. We cannot rule out that positions 9 and 25 may also be adapting to a substrate binding in the same position, because they could influence the overall conformation and thus binding specificity of the chromo domain. However, positions 9 and 25 are expected to be solvent accessible on the opposite side of the Rhino chromo domain and may represent an additional, potentially novel, interaction surface.

**Table 2:**
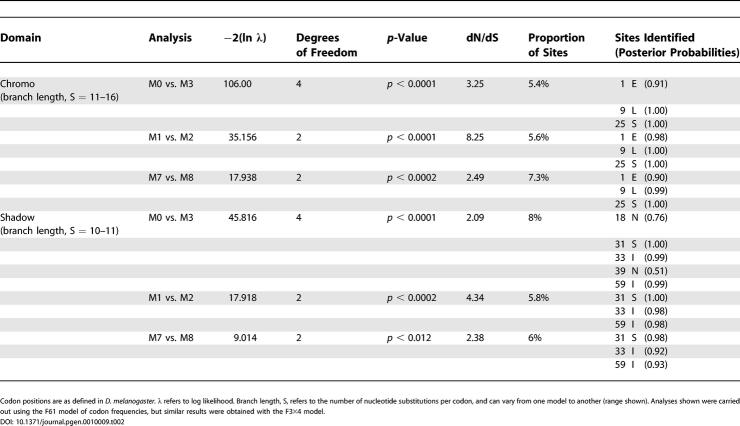
PAML Analyses of *rhino* Chromo and Shadow Domains in *Drosophila*

Codon positions are as defined in *D. melanogaster.* λ refers to log likelihood. Branch length, S, refers to the number of nucleotide substitutions per codon, and can vary from one model to another (range shown). Analyses shown were carried out using the F61 model of codon frequencies, but similar results were obtained with the F3×4 model.

We have already shown that the shadow domain is under strong positive selection between *D. melanogaster* and *D. simulans*. To find out if some codons of the shadow domain have also been under continuous positive selection, we carried out a PAML analysis. A tree based on the shadow domain amino acid alignment also recapitulates *Drosophila* phylogeny (Figure 7B). We found significant evidence for positive selection, with most of the signal coming from just three codons (Table 2; Positions S31, I33, and I59 in Figure 7B). On the structure of a mouse HP1β shadow domain dimer [46], positions 31 and 33 are on the same side of the shadow dimer and should be available for protein–protein interactions. The vertebrate HP1 chromo shadow domain dimerization site is known to bind to many proteins through their PxVxL motif [47–49]. It is unclear if these interactions are conserved in Rhino, but rapid evolution in this region (including position 59 identified in our PAML analysis) certainly has the potential to easily influence protein–protein interactions.

PAML analyses like these are very useful to highlight codons that have been repeatedly subject to positive selection [43,50]; however, they do run a risk of false positives. This is somewhat ameliorated in our dataset because the tree lengths are of moderate value (Table 2). Similar tree lengths have been shown by simulations to have a significantly lower risk of false positives [51]. Nonetheless, the true test for the significance of these positively selected residues will come from functional assays on Rhino function and localization.

## Discussion

In this paper, we have undertaken an evolutionary study of HP1-like proteins, with the ultimate aim of discerning the selective pressures that act on heterochromatin. We have found that Rhino, the only HP1 paralog that is expressed predominantly in ovaries, encodes a protein that has a unique localization pattern in S2 cells. Although it is excluded from the euchromatic compartment, the Rhino protein does not overlap with HP1A or H3K9me. This immediately suggests that H3K9me or HP1A does not mark all *Drosophila* heterochromatin, and that Rhino has a uniquely different specificity for a previously unappreciated compartment in heterochromatin.

It has not been easy to discern the molecular function of *rhino* from mutant phenotypes in eggshell defects. Possibilities range from a role for Rhino in gross chromatin structural changes, to transcriptional or translational regulation and even microtubule organization in the oocyte [36]. Despite our current lack of knowledge about the molecular function of *rhino*, the fact that mutations are female sterile, point to its importance to proper oogenesis [36]. *HP1A,* which is far better understood, is an essential gene. Such chromosomal proteins serving crucial functions are expected to be under strong evolutionary constraints and purifying selection. Although this is true for four of the five *D. melanogaster* HP1s including HP1A (see Figure 4), we find that all three domains of *rhino* have evolved under positive selection using multiple criteria, including dN/dS ratios, McDonald-Kreitman, and PAML analyses [39,43]. What could be driving this positive selection of such an important HP1 protein?

Can co-evolutionary pressures explain the positive selection acting on *rhino*? For instance, *rhino* might be continually “catching up” to mutations in interacting proteins required for its function. We believe this is unlikely, because mutations that compromise a required interaction are likely to be culled out of the population by purifying selection, long before a chance compensatory mutation in *rhino* can occur. A second possibility is that the positive selection of *rhino* may be driven by changes in the regulation of key genes between two species. Although we cannot formally rule out such a scenario, it appears unlikely to explain the relatively constant positive selection that we have seen for approximately 25 million years of *Drosophila* evolution.

Positive selection need not involve *rhino'*s “normal” function, whatever that may be, but rather underlie a second and unrelated “defense” function of *rhino*. In such a scenario the positive selection on *rhino* would be driven by a recurrent intracellular conflict that yields a selection advantage to the “winner.” Genes encoding proteins involved in direct host–parasite interactions are often subject to positive selection. In this case, changes that are beneficial for the parasite (to evade interactions for instance) will be followed by selection favoring changes in the host proteins (that restore interactions). Thus, two antagonistic entities locked in genetic conflict face repeated episodes of positive selection, only to arrive at the same quasi-steady state, a scenario formalized as the “Red Queen” hypothesis [52]. *rhino* may be subject to the same kind of genetic conflict that occurs intracellularly. It is especially intriguing that the only HP1 we have found to be subject to positive selection is expressed predominantly in ovaries ([36] and Figure 2), where such a competitive advantage has directly heritable consequences. We consider two models of genetic conflict to explain *rhino'*s positive selection.

Under the first model, *rhino* participates in suppressing “selfish” behavior of centromeres, which can compete to maximize their transmission advantage in female meiosis, where only one of four meiotic products is destined to become the egg [21]. We have previously proposed that this kind of drive can have deleterious consequences for male meiosis and is likely to be suppressed either by centromeric proteins altering their DNA-binding specificity [19,20] or by heterochromatin proteins evolving to limit centromere boundaries, and thereby limiting “strength” [4,21,53]. Similar selective pressures have been previously proposed to result in deleterious mutations in the *nod* chromokinesin in *D. melanogaster* [54]. *rhino* may represent another repressor of the drive by directly or indirectly influencing centromere strength.

A second model is that positive selection on *rhino* is a direct result of genetic conflict between *rhino* and mobile genetic elements. Although we have no evidence to support this hypothesis, it is attractive for several reasons. Transposable elements can evolve rapidly and differ significantly between *Drosophila* species, including *D. melanogaster* and *D. simulans* [55,56]. Rhino-GFP localizes to the heterochromatic region of the nucleus (see Figure 3), which is highly enriched in transposable elements [57]. Finally, genome-bound transposable elements can only increase their genomic copy number by transposing in the germline, increasing selective pressures on host proteins that act as suppressors of germline transposition. Rhino may either interact with the integration machinery of transposons to direct their integration into transcriptionally silent heterochromatin, or it may directly bind and transcriptionally repress transposable elements that are newly introduced into heterochromatin. Some transposable elements are known to be major in vivo targets of HP1A, apparently involving the RNAi (RNA interference) pathway [1,11,58–62]. Similarly, *rhino* may be under continual selection to directly bind transposable elements.

Whatever is driving the positive selection of *rhino,* mutations in any of Rhino's three domains appear to be selected to give *rhino* the upper hand in the current round of competition. The chromo and related shadow domains are very versatile interaction domains that can influence binding to DNA, RNA, and proteins [63]. The hinge domain can also strongly influence localization of HP1-like proteins [64,65]. Future experiments will address the functional role of the three amino acids under recurrent positive selection in the chromo and shadow domains (Figure 7) and help to distinguish between our models of what drives the positive selection of *rhino*. These experiments promise to reveal insights into the organization of a substantial portion of *Drosophila* genomes. It is probably not a coincidence that we have found positive selection only in an HP1-family member that is expressed predominantly in ovaries. Indeed, a restricted expression pattern may have allowed detection of a previously unremarked conflict that shapes at least a fraction of *Drosophila* heterochromatin, via the positive selection of *rhino*. Such a signal may have been masked for other HP1s due to their constrained roles in other tissues.

Our results complement previous findings that other proteins that bind heterochromatin appear to be among the most rapidly evolving proteins in an unbiased screen in *Drosophila* [67–68], although this does not appear to be the result of positive selection [69]. Polymorphisms in heterochromatin-binding proteins can have direct effects on non-disjunction frequencies [54,70,71]. Similarly, although HP1A, -B, and -C appear to be conserved and evolving under purifying selection, HP1 evolution (in both sequence and gene copy number; see Figure 1) in general appears quite rapid for a chromosomal protein with a highly conserved function in most eukaryotes. Thus, rapid changes in the genomic landscape may underlie rapid diversification of genes encoding HP1s and chromosomal proteins in general.

## Materials and Methods

### 

#### Sequences from *Drosophila* and databases and RT-PCR.

*Drosophila* species and strains (Table S1) were obtained from the *Drosophila* stock center (currently in Tucson, Arizona) and genomic DNA was prepared by standard methods [19]. The *rhino* locus was amplified using PCR Supermix High Fidelity (Invitrogen, Carlsbad, California, United States) with the primers indicated in Table S2. PCR products were either sequenced directly or following Topo-TA cloning (Invitrogen). RNA was prepared from whole male or female flies or different tissues (head, ovary, testis, or carcass) using a kit (Qiagen RNeasy; Qiagen, Valencia, California, United States) and cleared of genomic DNA by DNase I digestion (Ambion DNA-free; Ambion, Austin, Texas, United States). RNA concentrations were measured from various tissues, and the same amount of total RNA was used as template in the RT-PCR analysis. RT-PCR (Invitrogen) to evaluate the presence of *rhino* mRNA was carried out using Dmid1f and Dmid2b primers (Table S2) that span the *rhino* intron, along with *actin-42A* primers [72] as a control. For *D. bipectinata*, primers dv15 and dv230 that span the *rhino* intron were used. RT-PCR and sequencing was carried out to confirm the predicted splice-site positions for *rhino* from *D. simulans* (strain 2), *D. bipectinata,* and *D. miranda*. Splice sites for *rhino* from other species were predicted using Berkeley Drosophila Genome Project Splice site predictor (http://www.fruitfly.org/seq_tools/splice.html). All sequences have been deposited in Genbank (accession numbers AY944308–AY944358, Table S2).

#### Sequence analysis.

Sequences were assembled using DNA Strider [73]. Clustal_X [74] was used to obtain pairwise or multiple alignments and to generate formatted files for further analysis. Pairwise sequence alignments used for dN/dS analysis were hand edited, using the amino acid sequence as a guide to place indels. For instance, there is an 80 amino acid length difference between the *D. melanogaster* and *D. simulans* hinge regions. These regions cannot be compared in tests for positive selection. Pairwise dN and dS comparisons and confidence values were calculated using the K-estimator software [75,77]. Sliding window size was arbitrarily chosen as 100 bases with 35 base steps for all pairwise dN/dS comparisons. Confidence interval estimates were calculated using Monte Carlo simulations, taking into account (1) dN and dS values, (2) the number of codons, (3) transition: transversion ratio, and (4) GC content and amino acid composition. Thus, K-estimator [75] at least takes into account most of the confounding variables that are known to give false positives in terms of dN/dS. We also present a dN/dS analysis using the reconstructed hypothetical ancestors to all the *D. melanogaster* and *D. simulans* *rhino* sequences (Figure S2).

The DNASP software package [77] was used to perform several tests for positive selection using genomic sequence of *rhino* from 17 strains of *D. melanogaster* and 11 strains of *D. simulans*. The Fu and Li [40], Tajima's D [41], and Hudson-Kreitman-Aguade [42] tests were carried out on the complete sequence, including the intron, whereas the McDonald-Kreitman test [39] was carried out on coding regions only (1,209 total positions with indels removed). Fixed replacement changes in the chromo and chromo shadow domains were polarized using *D. yakuba* and *D. teissieri* sequences as outgroups, but we could not unambiguously polarize all changes in the hinge region. The expected fixed replacement changes (Rf_expected_) shown in Figure 4B were calculated from the ratio Rf_expected_ = Sf_observed_(Rp_observed_/Sp_observed_) according to the neutral expectation in the McDonald-Kreitman test, where R = replacement, S = synonymous, f = fixed between population, p = polymorphic within the population (similar to the previously proposed “Neutrality Index” [78]). A sliding window of 300 nucleotides with step size of 25 was used for presentation purposes.

Neighbor-joining phylogenetic trees were constructed using the PAUP software, version 4.0b10 [79] and appropriate Clustal_X multiple alignments of either the chromo or chromo shadow domains. A total of 1,000 replicates were carried out for bootstrapping. Maximum likelihood analysis was performed with the PAML software package [43] in separate analyses for multiple alignments of the chromo domain and the shadow domains (the rapid evolution of the hinge in both size and sequence precluded its comparison in such a multiple alignment). Codons that were repeatedly subject to positive selection were identified using N sites models (M1, M7) that do not permit positive selection compared to models (M2, M8) that permit sites to evolve under positive selection. The strength of positive selection was calculated by comparing twice the log likelihood difference (M2 vs. M1, M8 vs. M7) in a chi-square test with two degrees of freedom. Codons that were identified as having evolved under positive selection with high posterior probabilities (*p* > 0.95) were highlighted on a three-dimensional structure of the respective domains and visualized using the Cn3D software (version 4.0) [80].

#### Plasmid constructs.

A plasmid for expressing *rhino* as a C-terminal GFP fusion protein under control of the hsp70 heat shock promoter (HSRhiGFP) was constructed as follows: *rhino* coding sequence flanked by XbaI and NotI restriction enzyme sites was amplified by RT-PCR from *D. melanogaster* (Canton S) using primers KcRhiF and KcRhiB (Table S1). The PCR product was digested and cloned into a modified heat shock expression plasmid [81] that had been digested with XbaI and EagI and phosphatase treated to yield the *rhino* open reading frame followed by a six amino acid linker and GFP. Correct cloning was verified by sequencing. An N-terminal fusion protein of a biotin recognition peptide (MAGGLNDIFEAQKIEWHEDTGGS) to *rhino* (BLRPRhi) was constructed as follows: Primers dv99 and dv100 were used to amplify *rhino* coding sequence with flanking NotI and BamHI restriction enzyme sites from the HSRhiGFP plasmid. The PCR fragment was TA cloned and the sequence verified before digestion of the TA clone and subcloning of the gel-isolated fragment into a BLRP expression vector with a metallotheionine promoter [82,83]. A plasmid (pBirA) expressing the *Escherichia coli* biotin ligase enzyme (BirA) from a metallotheionine promoter was a gift from Takehito Furuyama.

#### Cell culture, transfection, and immunostaining.

S2 cells (Invitrogen, D-mel2) were maintained in serum-free insect media (Invitrogen) supplemented with 90 ml/l of 200 mM L-Glutamine (Sigma, St. Louis, Missouri, United States). Twenty micrograms of the HSRhiGFP plasmid was transfected as previously described [81]. Cells were heat shocked for 1 h on the next day and allowed to recover for 2 h before immunostaining [84]. In the case of the BLRPrhino construct, 10 μg of plasmid DNA were co-transfected with 10 μg of pBirA plasmid that contains the biotin ligase under control of a metallotheionine promoter. After overnight incubation, cells were induced for 3 h with 500 μM CuSO_4_, added directly to the media, followed by immunostaining. HP1A, HP1B, and HP1C antibodies have been previously described [35]. Antibodies to H3K9me or H3K4me were purchased from Upstate Biotech (Waltham, Massachusetts, United States). Monoclonal mouse anti-Fibrillarin antibody was purchased from Encor Biotechnology Inc (Alachua, Florida, United States). All antibodies, including the secondary Texas-red fluorescently labeled goat anti-rabbit or anti-mouse antibodies (Amersham, Piscataway, New Jersey, United States), were used at a dilution of 1/200, with the exception of the anti-fibrillarin antibody that was used at 1/500. Images of nuclei were obtained and de-convolved using the Deltavision software (Applied Precision, Issaquah, Washington, United States).

## Supporting Information

Figure S1 Rhino-GFP Localization in *Drosophila* S2 Cells These additional images of Rhino-GFP show a localization pattern that is distinct from HP1A, H3K4me, and H3K9me. In addition, an N-terminal biotinylated-tagged Rhino protein shows the same localization pattern as that of the C-terminal GFP-tagged Rhino protein.
(5.2 MB PDF)

Figure S2 A Sliding Window dN/ dS Analysis Only those changes that were found to have been fixed differences between *D. melanogaster* and *D. simulans* were used*.* All intraspecific polymorphisms were eliminated for this analysis. Compared to Figure 4, the signal for positive selection now appears concentrated exclusively in the C-terminal region of *rhino*.
(203 KB PDF)

Table S1 All Polymorphisms within the Coding Region of the *rhino* Gene in *D. melanogaster* and *D. simulans* Are Shown Changes are highlighted as being either fixed (f) between species or polymorphic (p) within species, as replacement (R) or synonymous (s) changes. Fixed changes were polarized using an outgroup species to changes along either the *D. melanogaster* (m) or *D. simulans* (s) lineages. Many changes could not be unambiguously polarized.
(34 KB DOC)

Table S2 List of Primers Used and Accession Numbers of Sequences Obtained in This Study (36 KB XLS)

### Accession Numbers

The Flybase (http://flybase.bio.indiana.edu) accession numbers of the genes discussed in this paper are *rhino* (CG10683) and *HP1E* (CG8120). New sequences obtained during the course of this study have been deposited in Genbank under the accession numbers AY944308–AY944358. The Molecular Modeling Database (MMDB; http://www.ncbi.nlm.nih.gov/Structure/MMDB/mmdb.shtml) accession numbers of the proteins discussed in this paper are H3K9me (19011, PDB 1KNE) and HP1****β shadow domain dimer (13286, PDB 1DZ1).

## Abbreviations

dN: number of replacement changes per site
dS: number of synonymous changes per site
f: fixed between species
GFP: green fluorescent protein
HP1: heterochromatin protein 1
H3K4me: histone H3 dimethylated at lysine 4
H3K9me: histone H3 dimethylated at lysine 9
p: polymorphic within species
R: replacement
S: synonymous
