# Supplementary figures and images for: Positive Selection Drives the Evolution of rhino, a Member of the Heterochromatin Protein 1 Family in Drosophila
Source: PLoS Genet. 2005 Jul 25;1(1):e9. doi: 10.1371/journal.pgen.0010009 (PMC1183528; doi:10.1371/journal.pgen.0010009)

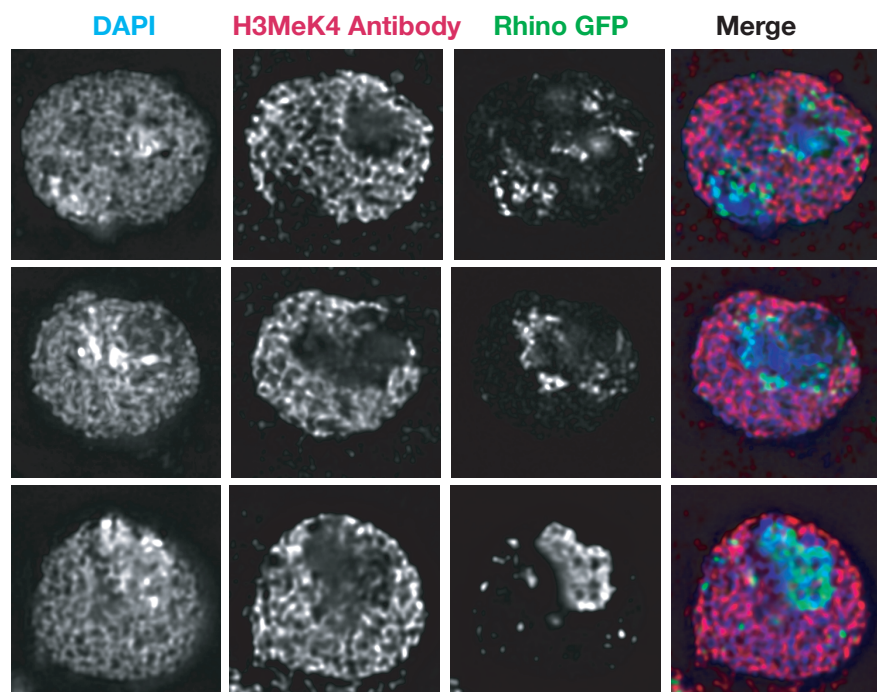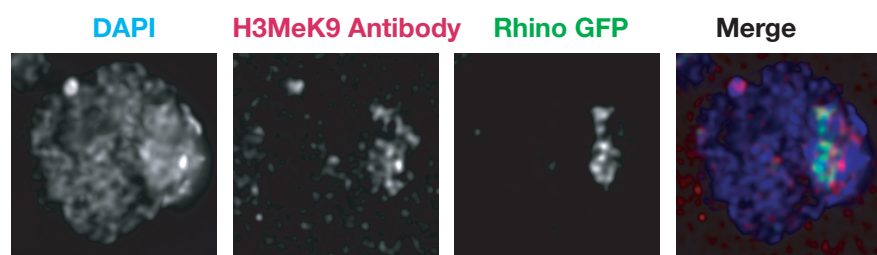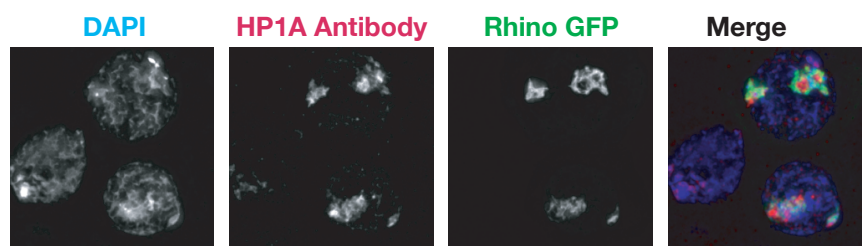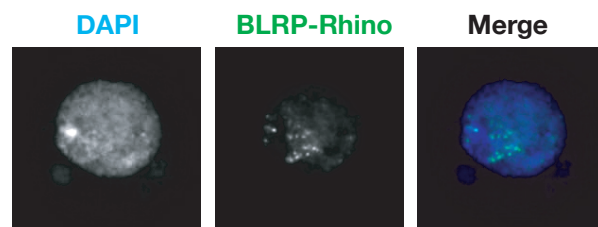

Vermaak et al, Supplementary Figure 1

Supplement: Figure S1 — These additional images of Rhino-GFP show a localization pattern that is distinct from HP1A, H3K4me, and H3K9me. In addition, an N-terminal biotinylated-tagged Rhino protein shows the same localization pattern as that of the C-terminal GFP-tagged Rhino protein. (5.2 MB PDF) [file pgen.0010009.sg001.pdf]

dN/dS using only fixed interspecies changes between *D. melanogaster* & *D. simulans*

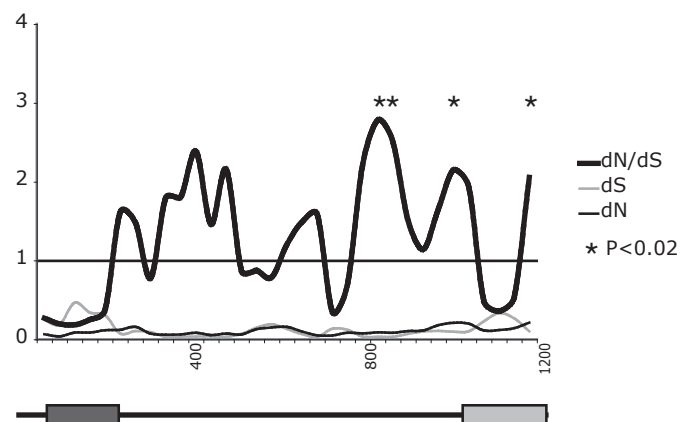

Supplement: Figure S2 — Only those changes that were found to have been fixed differences between D. melanogaster and D. simulans were used. All intraspecific polymorphisms were eliminated for this analysis. Compared to Figure 4, the signal for positive selection now appears concentrated exclusively in the C-terminal region of rhino. (203 KB PDF) [file pgen.0010009.sg002.pdf]
