# Supplementary material for: Positive Selection Drives the Evolution of rhino, a Member of the Heterochromatin Protein 1 Family in Drosophila
Source: PLoS Genet. 2005 Jul 25;1(1):e9. doi: 10.1371/journal.pgen.0010009 (PMC1183528; doi:10.1371/journal.pgen.0010009)
Supplement: Table S1 — Changes are highlighted as being either fixed (f) between species or polymorphic (p) within species, as replacement (R) or synonymous (s) changes. Fixed changes were polarized using an outgroup species to changes along either the D. melanogaster (m) or D. simulans (s) lineages. Many changes could not be unambiguously polarized. (34 KB DOC) [file pgen.0010009.st001.doc]

Supplementary Table 1

Position 2382148418969678805628232633567376462923960343681262462383021243857893538256795691493451524782565690

1114555678899333455566789923333445779900017778880002224445789990012223345666667778889960011223334445

111111111111122222222222233333333334444444444444445555555555555555555555556666666666666

cnbw CCGTTTC**GATTAGAGCACACCATACAG**TCATGGTTAGTTCGCGGGATAGAACGAGATCGTATCTGACAAAAGACTTCGTGCTTCCTAAAGAAACCCCCCT

Mel0 .................................................................C..................................

Mel2 ....................................................................................................

Mel3 ...................................................A................................................

Mel4 .........................................A.....G.........AT......G...........................A..T..C

Mel6 ....................................................................................................

Mel7 ....................................................................................................

Mel5 .....C..........................................................................T...................

MelC8 .....C...........................................................................G..................

MelC13 .....C..................................................................T...........................

MelC18 .....C...........................................................................G..................

MelC26 .....C...........................................................................G..................

MelC159 .....C.........................................G....................................................

MelC194 .....C.................................A..T....G....A...................T...........................

MelC216 .....C....................................T....G....A...............................................

MelZim159 .........................................A.....G.........AT.........................................

M346 .....C...................................A.....G.........AT......G...........................A..T..C

Sim1 AAA.GC.CTCAGCCATGTGTAGACTCCGATGTTCCCACA.A..AA.CGAGG..CC.G..GGCTCA.....GATG...ACT..GTGCGGCACT.....G..

Sim6 AAA.GC.CTCAGCCATGTGTAGACTCCGATGTTCCCACA.A..AA.CGAGG..CC.G..GGCTCA.....GATG...ACT..GTGCGGCACT.....G..

Sim8 AAA.GC.CTCAGCCATGTGTAGACTCCGATGTTCCCACA.A..AA.CGAGG..CC.G..GGCTCA.....GATG...ACT..GTGCGGCACT.....G..

Sim9 AAA.GC.CTCAGCCATGTGTAGACTCCGATGTTCCCACA.A..AA.CGAGG..CC.G..GGCTCA.....GATG...ACT..GTGCGGCACT.....G..

Sim2 AAAAGC.CTCAGCCATGTGTAGAC.CCGATGTTCCC.CA.A..AAGCGAGG..CC.G..GGCT.A.....GATG.ATACT..GTGCGGCACT..AA.GG.

Sim4 AAA.GC.CTCAGCCATGTGTAGAC.CCGATGTTCCC.CA.A..AA...AGG...C.G...GCTCA.....GATG...ACT..GTGCGGCACT.....G..

Sim5 AAA.GC.CTCAGCCATGTGTAGAC.CCGATGTTCCC.CA.A..AA...AGG...C.G..GGCTCA.....GATG...ACT..GTGCGGCACT.....G..

SimC11 AAA..CACTCAGCCATGTGTAGAC.CCGATGTTCCC.CA.A..AA.CGAGG..TCCG..GGCT.A...T.GATG...ACT..G.GCGGCACT.....G..

Sim162 AAAAGC.CTCAGCCATGTGTAGAC.CCGATGTTCCC.CA.A..AAGCGAGG..CC.G..GGCT.A.....GATG.ATACT..GTGCGGCACT..AA.GG.

Sim175 AAAAGC.CTCAGCCATGTGTAGAC.CCGATGTTCCC.CA.A..AA..GAGG...C.G..GGCTCA.....GATG.ATACT..GTGCGGCACT.....G..

Sim169 AAA.GC.CTCAGCCATGTGTAGAC.CCGATGTTCCC.CA.A..AA...AGG...C.G..GGCTCA.AC.CGATGA..ACT..GTGCGGCACTT....G..

f/p/b fffpppp**fffffffffffffffffpff**fffffffffpffpfppffpppfffpppfpfpppfffpfpppppffpfpppfffppfpffffffffpppppfpp

R/s RRsRssRRRsRssRRsRsRsssRssRRRRRsRRRRsRRRssRRRRRRRRsRRRRRRRsRRRsRRRRRsRRRsRRRRRRRssRRRRsRRRsRRRRRssRRR

m/s/?/- ??? ms?s?m?m??ssmsmmm mmm??mmsm?m ss m mm mms m m mms ? ss s mss m msmmm?mm -

Position 5798043404024501429468456792456737160302239052684569024902334135348037479803690517134701687804836795

5668990011333344467888112445555566790022333445556666777788899000111334555688889900333344446677789990

6666667777777777777777888888888888889999999999999999999999999000000000000000000011111111111111111112

111111111111111111111111111111111111111

cnbw GCGCTTCTGACGGCGTTTTCTCAAGAGCGCCAATTCTTGATGCGGAGCCCGCCCAAAAAGGATCTG**AGATAATCTCACATGGGATTTGTAGAAAAGTCTC**

Mel0 .....................................................A.....AA....A....G.............................

Mel2 .............................T......................................................................

Mel3 ....................................................................................................

Mel4 ....................A......G.........................A.....AA....A....G.............................

Mel6 ....................................................................................................

Mel7 ....................................................................................................

Mel5 ................................T....................A.....AA....A....G.............................

MelC8 ...........................G...........C.............A.....AA....A....G..........................T..

MelC13 ...........................G.........................A.....AA....A....G.............................

MelC18 ...........................G.A.........C.............A.....AA....A....G..........................T..

MelC26 ...........................G.A.........C.............A.....AA....A....G..........................T..

MelC159 A..........................................................AA....A....G.............................

MelC194 .....................................................A.....AA....A....G.............................

MelC216 ...........................................................AA....A....G.............................

MelZim159 .....................................................A.....AA....A....G.............................

M346 ....................A......G.........................A.....AA....A....G.............................

Sim1 .GAA..A.TG.AATTCCAAT.TGG..AGA....GAA.GA.CATA..AG..T.G..TTGG..GAGCTGTGG.GATAGCTCCAA.CCGAT.GAGGGTCC.CT

Sim6 .GAA..A.TG.AATTCCAAT.TGG..AGA....GAA.GA.CATA..AG..T.G..TTGG..GAGCTGTGG.GATAGCTCCAA.CCGAT.GAGGGTCC.CT

Sim8 .GAA..A.TG.AATTCCAAT.TGG..AGA....GAA.GA.CATA..AG..T.G..TTGG..GAGCTGTGG.GATAGCTCCAA.CCGAT.GAGGGTCC.CT

Sim9 .GAA..A.TG.AATTCCAAT.TGG..AGA....GAA.GA.CATA..AG..T.G..TTGG..GAGCTGTGG.GATAGCTCCAA.CCGAT.GAGGGTCC.CT

Sim2 .GAA..A.T..A.TTC..AT.TGG..AGA....GAAGGA.CATA..AG..T.G..TTGG..GAGCTGTGG.GCTAGCTCC...CCGATAGAGGGTCC.CT

Sim4 .GAA..A.TG.AATTCCAAT.TGGA.AGA..G.GAA.GA..ATAAGAGT.T.G..TTG...GAGCTGTGG.GCTAGCTCC..ACCGATAGAGGGTCC.CT

Sim5 .GAA..A.TG.AATTCCAAT.TGGA.AGA..G.GAA.GA..ATAAGAGT.T.G..TTG...GAGCTGTGG.GCTAGCTCC..ACCGATAGAGGGTCC.CT

SimC11 .GA..AACT.GA.TTC..AT.TGG.GAGA....GAA.GA..ATA..AGT.T.G..TTG...GAGCTGTGG.GCTAGCTCC...CCGATAGAGGGTCC.CT

Sim162 .GAA..A.T..A.TTC..AT.TGG..AGA....GAAGGA.CATA..AG..T.G..TTGG..GAGCTGTGG.GCTAGCTCC...CCGATAGAGGGTCC.CT

Sim175 .GA.C.A.T..A.TTCC.AT.TG...AGA.AG.GAA.GA..ATA..AGTGTGG.CTTC...GAGCTGTGG.GCTAGCTCC...CCGATAGAGGGTCC.CT

Sim169 .GAA..A.TG.AATTCCAAT.TGGA.AGA..G.GAA.GA..ATAAGAGT.T.G..TTG...GAGCTGTGG.GCTAGCTCC..ACCGATAGAGGGTCC.CT

F/p/b pffpppfpfppfpfffppffpffpppfpfppppfffpffppfffppffppfpfppffppppffffp**ffffpfbfffffffpppfffffpffffffffpff**

R/s RRsRsRRRRsRsRsRsssRRsRRRRRssRRsRRRRRsRRRRRsRsRRRRRRsRsRRRRRsRRRRRsRRRRRRssRRssRssssRsRRRsRsRsRRRRsRR

m/s/?/- -- - - - --- -- -- - - --- -- --- -- - m ?? mmm? ?mmm mmss?msms msmms ssssssms ms
